# Optical Tip Clearance Measurements as a Tool for Rotating Disk Characterization

**DOI:** 10.3390/s17010165

**Published:** 2017-01-15

**Authors:** Iker García, Joseba Zubia, Josu Beloki, Jon Arrue, Gaizka Durana, Gotzon Aldabaldetreku

**Affiliations:** 1 Department of Communications Engineering, E.T.S.I. of Bilbao, University of the Basque Country UPV/EHU, Alda. Urquijo s/n, Bilbao 48013, Spain; joseba.zubia@ehu.eus (J.Z.); jon.arrue@ehu.eus (J.A.); gaizka.durana@ehu.eus (G.D.); gotzon.aldabaldetreku@ehu.eus (G.A.); 2 CTA, Aeronautical Technologies Center, Bizkaia Technological Park, Zamudio 48170, Spain; josu.beloki@ctabef.com; 3 Department of Nuclear Engineering and Fluid Mechanics, E.T.S.I. of Bilbao, University of the Basque Country (UPV/EHU), Bilbao 48013, Spain

**Keywords:** optical fiber sensor, rotating disk, tip clearance, vibration measurement, nodal diameter

## Abstract

An experimental investigation on the vibrational behavior of a rotating disk by means of three optical fiber sensors is presented. The disk, which is a scale model of the real disk of an aircraft engine, was assembled in a wind tunnel in order to simulate real operation conditions. The pressure difference between the upstream and downstream sides of the disk causes an airflow that might force the disk to vibrate. To characterize this vibration, a set of parameters was determined by measuring the tip clearance of the disk: the amplitude, the frequency and the number of nodal diameters in the disk. All this information allowed the design of an upgraded prototype of the disk, whose performance was also characterized by the same method. An optical system was employed for the measurements, in combination with a strain gauge mounted on the disk surface, which served to confirm the results obtained. The data of the strain gauge coincided closely with those provided by the optical fiber sensors, thus demonstrating the suitability of this innovative technique to evaluate the vibrational behavior of rotating disks.

## 1. Introduction

The operation of multiple devices, such as computer hard disks or circular saws, is based on rotating disks [1]. During operation, these rotating disks may experience vibrations that reduce their performance and service lives. Such vibrations could be due to several reasons, e.g., tolerances in the manufacturing process, aging, or some asymmetry in the disk. Vibrations of critical amplitudes cause a reduction in the reliability of the devices, which may undergo failures due to fatigue [2]. Therefore, there is a great interest in exploring techniques that allow the monitorization and characterization of the vibrational behavior of rotating disks [3,4,5].

In this paper, we report on our evaluation works related to the vibrational behavior of a rotating disk that is a scale model of a real disk of an aircraft engine. Such evaluation was carried out by means of a non-contact method employing three optical fiber sensors (OFSs) [6,7]. Its interest lies in the fact that reliability of aircraft components is of capital importance, since a failure in one of them could lead to a catastrophic situation. Although it is completely unfeasible to totally avoid the chance of disk failures, nowadays the number of disk flaws has been considerably reduced by means of melting-process controls and non-destructive-inspection techniques [8]. Up to now, these have usually been carried out employing strain gauges, which still constitute the most common sensors for the characterization of the strain and vibrations of aircraft components. However, they present several drawbacks, such as the need of a telemetry system to transmit the signals, or their complex and time-consuming wiring. In comparison, OFSs present intrinsic benefits [9,10,11], so they are being more and more used to evaluate the structural health of aeronautical components [12]. One of them is the engine. In previous works, we developed a sensor for it that was based on a bundle of optical fibers, which served to carry out tip-clearance (TC) measurements in low pressure turbines [13] and compressors [14] of aircraft engines. The TC is the gap between the tip of the turbine/compressor blades and the casing of the engine, and it is a crucial parameter related to the efficiency of the engine [15]. This parameter has also been employed to evaluate other phenomena, such as cracks in the blades [16]. To characterize the vibrational behavior of our special rotating disk, we decided to employ three OFSs, because, as a matter of fact, the distance from the edges of a rotating disk to the casing of a wind tunnel can be interpreted as a clearance that varies as a result of disk vibrations. This method based on three OFSs provided a great reduction in the time needed to get the system ready to start the tests in comparison to a system based only on strain gauges. In addition, as only one strain gauge is required, there is a lower influence in the mechanical behaviour of the disk with respect to a measuring system based on multiple strain gauges.

The disk was assembled in a wind tunnel to assess its vibrational behavior. In order to simulate real operation conditions, a pressure difference was set between the upstream and downstream sides of the disk. This, in turn, creates an air flow through the gap between the casing of the wind tunnel and the disk edge. Under particular conditions, the air flow can force the disk to vibrate. This aerolastic instability, known as flutter, is self-initiated and also self-maintained, because the vibrations return energy to the disk in a way that their amplitudes increase dramatically if the damping is not able to dissipate that energy [17]. All details about the assembly of the disk in the wind tunnel and the OFSs configuration are described in Section 2. In Section 3, the results obtained for the two prototypes of the disk tested in the wind tunnel are presented and discussed. Finally, the conclusions of the work are summarized in Section 4.

## 2. Experimental Set-Up

The experiments were carried out in the transonic wind tunnel at Aeronautical Technologies Center (CTA). The CTA’s rotating-turbine-test facility is a continuous transonic-flow-test bed with an atmospheric inlet/outlet. The level of pressure/vacuum, the temperature and the mass flow are individually regulated, so that Mach and Reynolds numbers can be independently modified. 

**Figure 1:**
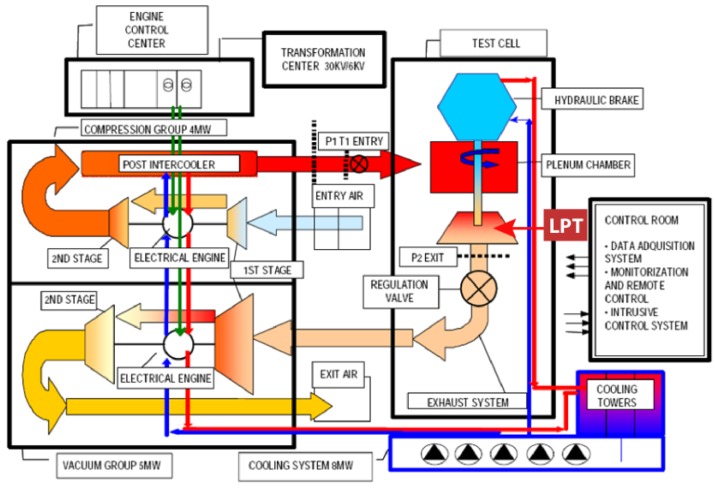
Scheme of the transonic test turbine facility. The disk under test was assembled in the position where the low-pressure turbine (LPT) is usually placed.

The supply and exit air conditions in the test section are achieved by employing two centrifugal compressor and vacuum groups, which are run, respectively, by electrical motors of 3.7 MW and 5 MW. The compressors are able to supply a maximum mass flow of 18 kg/s, with a maximum supply pressure up to 4.5 bar, a minimum exit pressure of 0.3 bar, and a temperature regulation from atmospheric temperature up to 160 °C. The turbine power is transmitted by a single shaft (up to 7800 rpm) to a dynamometer (up to 11,000 rpm 3.3 MW). The test section has a diameter of 1 m. A schematic of the facility is included in Figure 1.

The data acquisition system is able to acquire up to 800 pressure channels, 200 temperature channels and 28 vibration signals. It can also acquire 20 rotating signals and it incorporates high accuracy torque machines and duplicated rpm and mass flow measurements. More information regarding the tunnel can be found in [18].

**Figure 2:**
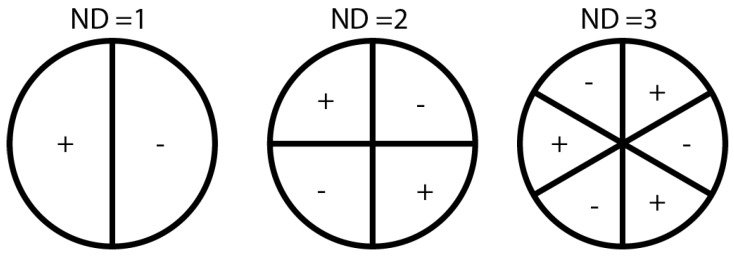
Disks vibrating with one, two and three nodal diameters, respectively.

As mentioned before, the main objective of the tests was to characterize the vibrational behavior of the rotating disk under test. Specifically, we needed to know the vibration frequencies and the number of nodal diameters (NDs) of the disk at those frequencies. A nodal diameter can be defined as the line of stationary points that separates parts of the disk vibrating out of phase with respect to each other. The concept of nodal diameter is represented in Figure 2, where we can see a disk vibrating with one, two and three nodal diameters.

This information enabled the design of an improved second prototype with damped vibrations. In order to obtain results from the disk that correspond to real working conditions, this was assembled in a wind tunnel where a pressure difference was established between both sides of the disk, ranging from 2 to 3 bar. As well as evaluating the case when the disk is not rotating, the disk’s performance was analyzed at its nominal rotating speed (RPM_nominal_), and at 1.5 and 2 times RPM_nominal_. In order to get these rotating speeds, the shaft was driven by a 100 kW electric motor and it was controlled by means of a hydraulic brake.

**Figure 3:**
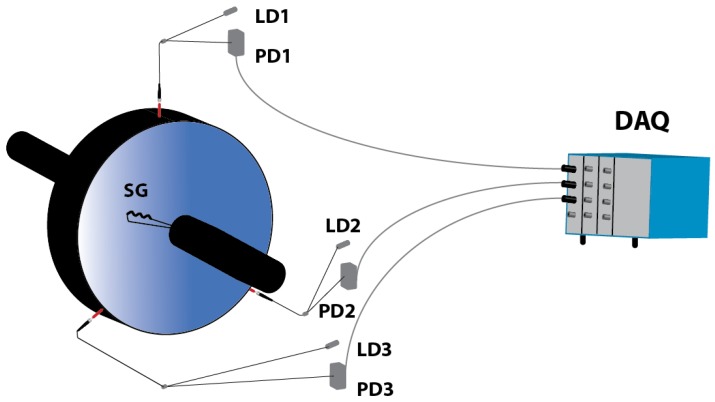
Experimental set-up for the TC measurements in the wind tunnel. LD x stands for Laser Diode x, PD x for Photodetector x, SG for Strain Gauge, and DAQ for Data Acquisition System.

Three OFSs were installed in the wind tunnel to carry out TC measurements. They were arranged at an angle of 120° between them and separated by 4.75 mm from the disk edge (see Figure 3). These reflective intensity-modulated OFSs were originally developed to perform TC measurements in turbines [19]. Their main component is a trifurcated bundle of optical fibers whose particular design allows us to transmit the light from a laser to the target, and to collect the reflected light which is employed to calculate the distance from the bundle tip to the target. All the components and the performance characteristics of the sensors have been described in [20]. Each of the three sensors provides two output signals, whose quotient serves us to obtain the distance to the target according to a calibration curve. This method provides immunity to the fluctuations of the light source, to changes in the reflectivity of the target surface, and to losses or misalignments between the sensor probe and the target [21]. Due to the high number of sensors and transducers needed to measure the temperature and pressure in multiple points of the wind tunnel, only three inputs of the acquisition system were available. In this situation, we have two options: (a) one sensor with two output signals; or (b) three sensors, each one with a single output, (see Figure 3). We chose the latter option because it implied a loss of accuracy of only about ±8% in the measured amplitude with respect to the first option. This loss of accuracy does not affect the detection of the vibration frequency, which was the main goal of the tests, since both options would yield the same frequency, although with different amplitudes. Besides, the use of three sensors allows us to calculate the eccentricity of the disk.

The probe fixed to the casing (Figure 3) does not detect the real vibration frequency of the disk when it is rotating, but a modulated frequency instead. The reason is that the measured frequency is modulated by the rotational speed and by the set of NDs excited in the disk, according to the following equation [22]:

(1) $$f_{os}=f_{disk}\pm f_{r}\cdot ND$$

where *f_os_* is the frequency detected by the OFS, *f_disk_* is the vibration frequency of the disk, *f_r_* is the rotational frequency, and *ND* is the number of nodal diameters. In order to check the ability of the sensors to detect the vibration frequency and to obtain the NDs of the disk, a strain gauge was instrumented on the disk surface.

**Figure 4:**
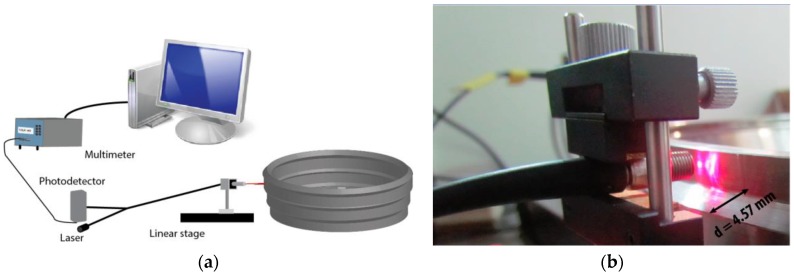
Sensor calibration process (**a**) and detail of the tip of the OFS illuminating the rotating disk during the calibration process (**b**).

**Figure 5:**
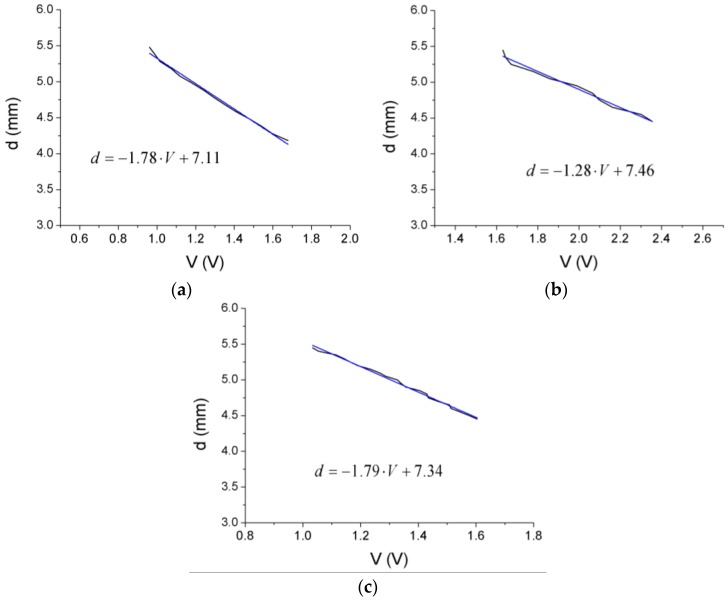
Calibration curve (black) and its linear fit (blue) for OFS 1 (**a**), OFS 2 (**b**) and OFS 3 (**c**) for the measuring interval.

The calibration process of the OFSs is illustrated in Figure 4. It is the flattest part of the disk that is illuminated, so as to get the most stable signal, taking into account that the disk will flutter. To obtain the calibration curve of each sensor, the values of the voltage of the photodetector were successively stored as the tip of the probe was separated from the disk surface in steps of 10 µm. This procedure was repeated three times to obtain a calibration curve in a range of distances up to 10 mm, and the results were averaged to obtain a more accurate calibration. Since the expected amplitude of the vibration was 0.25 mm, the resulting curve was linearized in the interval 4.5–5 mm to obtain the following calibration curves (represented in Figure 5):

(2) d=−1.78⋅V+7.11

(3) d=−1.28⋅V+7.46

(4) d=−1.79⋅V+7.34

where *d* is the distance to the disk (in mm) and *V* is the voltage of the photodetector (in V).

As can be seen in Figure 4b, the disk incorporates a flange whose depth is 4.57 mm. Therefore, to obtain the real TC of the disk, this distance must be subtracted from the value provided by Equations (2)–(4).

The signals of all the sensors involved in the tests were acquired using the dynamic-signal-acquisition module PXI-4472 from National Instruments. They were sampled at 25 k samples/s and the complete signal-acquisition process for each signal lasted for 5 s (125,000 samples). For the OFS, Matlab was used to obtain the Fast Fourier Transform (FFT) of each signal with a frequency resolution of 0.2 Hz. However, the FFT of the signal from the strain gauge was provided by the acquisition program employed in CTA with a frequency resolution of 2 Hz.

To determine the uncertainty of the measurements of the three OFSs, some tests were carried out in our laboratory. The tip of the probe was placed 4.5 mm away from the disk edge and it was moved outwards along 1 mm in 25-µm steps using a linear stage. In each step, the voltage of the photodetector was recorded. This procedure was repeated three times for each distance, and the standard deviation of the three measurements was calculated. The final uncertainty of each sensor (see Table 1) was obtained as the average of all the standard deviations for each distance.

## 3. Results

As mentioned in the previous section, the objective of the tests is to experimentally characterize the vibrational behavior of a rotating disk (first prototype) in a realistic operation condition. In this way, the manufacturer could optimize some parameters of the disk to reduce the vibrations in an upgraded design (second prototype). 

**Figure 6:**
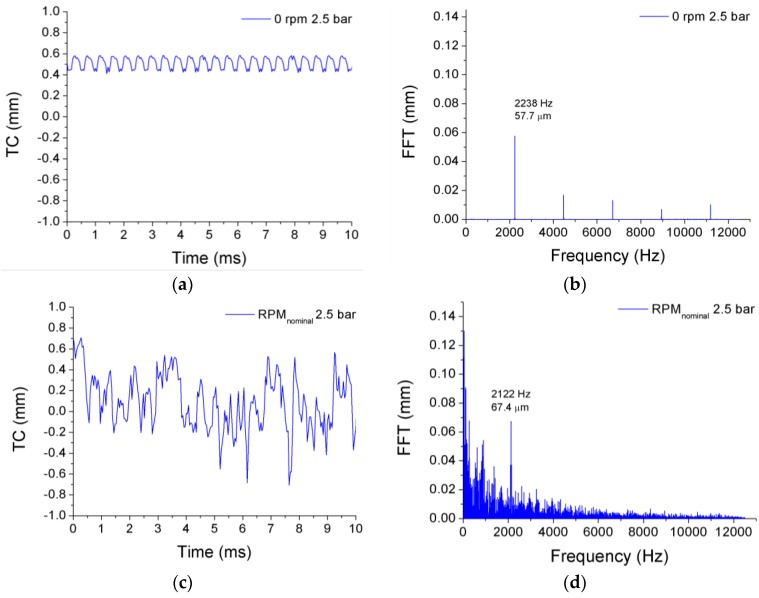
Time domain signals and their FFTs for OFS 1 when the disk is not rotating (**a**,**b**) and when it is turning at its nominal speed (**c**,**d**). Clear peaks appear at *f_os_* = 2238 Hz and *f_os_* = 2122 Hz, respectively.

Figure 6 shows the signals and their FFTs obtained for the case of the first prototype of the disk. The signals were obtained from OFS 1, which was placed on the upper part of the wind tunnel, in the direction of an imaginary vertical axis that passes by the center of the disk. OFS 2 and OFS 3 were placed forming angles of 120° and −120° with the direction of OFS 1, respectively. The graphs correspond to a pressure difference of 2.5 bar when the disk is rotating at nominal speed (c and d), and when it is not rotating (a and b). Notice that the airflow forces the disk to flutter even when it is static. Even in such a case, the TC varies substantially with the vibration of the disk, and the vibration frequency is easily identified by the peak in the FFT of the signal. The signal in the time domain becomes more complex when the disk is rotating, and its FFT shows that more vibration frequencies than in the static condition are detected.

**Figure 7:**
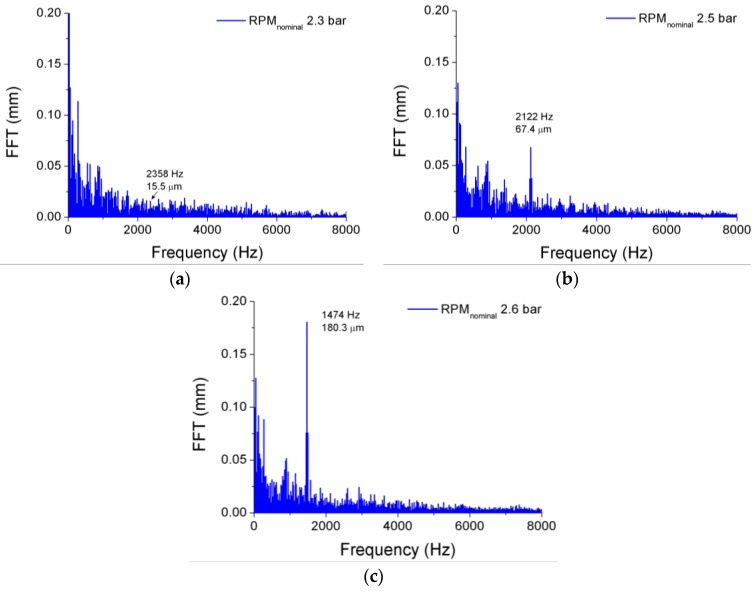
Evolution with the pressure difference of the vibration signal for the OFS 1 when the disk is rotating at RPM_nominal_.

The results obtained for the first prototype at various rotational speeds are summarized in Table 2, Table 3, Table 4 and Table 5. To calculate the amplitude of the vibrations, the TC of the disk is expressed in terms of its change, hence the negative values shown in Figure 6c. While the frequency of the strain gauge installed on the disk surface represents the real vibration frequency of the disk in every case, the frequency of the OFSs (*f_os_*) is modulated by the rotational speed of the disk and by the NDs. Thus, the vibration frequency of the disk provided by the OFS (*f_disk_*) has to be obtained by demodulating *f_os_* using Equation (1). The only exception is the case in which the disk is not rotating, since the frequencies of the signals from the strain gauge and from the OFSs coincide. All vibration frequencies provided by the OFSs match one another, and they also match those given by the strain gauge. The corresponding amplitudes are not correlated, since they correspond to vibrations measured at different points of the disk. For a fixed rotational speed, the amplitude of each vibration increases as the pressure difference between both sides of the disk becomes higher (see Figure 7). A situation that is worthy of study can be seen in Figure 7, in the case when the pressure difference is 2.6 bar and the disk is turning at RPM_nominal_. In such a case, the vibration frequency of the disk is markedly different, because it passes from 2247 Hz to 1574 Hz (2122 Hz to 1474 Hz for the frequency detected by the OFS). This is due to a change in the way this disk vibrates: for the lower pressure differences, the disk vibrates with five nodal diameters whereas at 2.6 bar the disk vibrates with ND = 4. The value of ND can be obtained from Equation (1), since *f_disk_* only matches the correct vibration frequency, which is given by the strain gauge, when the correct value of ND is used to demodulate *f_os_*. A similar behavior can be observed when the disk is rotating at 1.5 × RPM_nominal_, and at 2 × RPM_nominal_, with the peculiarity that it happens at lower pressure difference: 2.4 bar for 1.5 × RPM_nominal_ and 2.2 bar for 2 × RPM_nominal_.

All this information was employed to manufacture an improved design of the disk in a second prototype, which was also tested in the wind tunnel in order to evaluate the improvements in its vibrational behavior. The results of the tests for the second prototype are shown in Table 6, Table 7, Table 8 and Table 9. Again, all the frequencies provided by the OFSs and the strain gauge match each other for each rotational speed. The optimization of the design of the disk has allowed the achievement of a significant reduction in both the amplitude and the frequency of the vibration. The results for all the sensors can be seen in Table 10. When the disk is not rotating, there is a reduction of about 99% in the vibration amplitude and of 9.5% in its frequency. As the rotational speed increases, the vibration-amplitude reduction decreases down to 20% of the amplitude corresponding to the first prototype, whereas the frequency reduction remains constant at around 24%. In this table, the amplitude reduction for OFS 2 at 2 × RPM_nominal_ and 2.3 bar is negative, because, unexpectedly, an increase in the vibration amplitude for this sensor was measured under those conditions.

**Figure 8:**
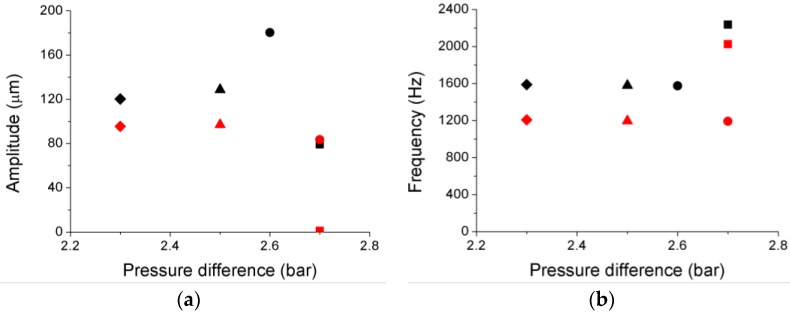
Vibration amplitude (**a**) and frequency (**b**) for the first Prototype (black) and the second Prototype (red) obtained by OFS 1 for the most similar working points (squares correspond to 0 rpm, circles to RPM_nominal_, triangles to 1.5 × RPM_nominal_ and diamonds to 2 × RPM_nominal_).

To better appreciate the improvement in the second prototype, the amplitude and frequency of the most similar working points for both prototypes have been depicted in Figure 8a,b. The results shown in both figures correspond to the OFS 1.

Another important improvement detected in the second prototype is the absence of changes in the way the disk was vibrating. There was no change in the number of nodal diameters of the disk. The second prototype vibrates with ND = 5 when the disk is not rotating and with ND = 3 for the rest of rotational speeds, independently of the rotational speed. Therefore, a more uniform and less complex vibrational behavior was achieved for this prototype.

The fact that three OFSs were employed allows us to determine the eccentricity of the disk. The circumference corresponding to the disk was calculated from the three TC measurements provided by each OFS when the disk is static. The lowest pressure difference (2.3 bar) was used to measure the eccentricity, so that the influence of the vibration in the measurement was minimum. The TC value was obtained as the mean value of the signal for an acquisition time of five seconds. The three TC points define a unique circumference, whose equation is easily calculated by solving the following system of equations to find A, B and C. Where *x_OFSi_* and *y_OFSi_* are the values obtained by subtracting the TC variation measurement of each sensor from the initial distance of the disk. 

(5) $$x_{OFS1}^{2}+y_{OFS1}^{2}+Ax_{OFS1}+By_{OFS1}+C=0$$

(6) $$x_{OFS2}^{2}+y_{OFS2}^{2}+Ax_{OFS2}+By_{OFS2}+C=0$$

(7) $$x_{OFS3}^{2}+y_{OFS3}^{2}+Ax_{OFS3}+By_{OFS3}+C=0$$

**Figure 9:**
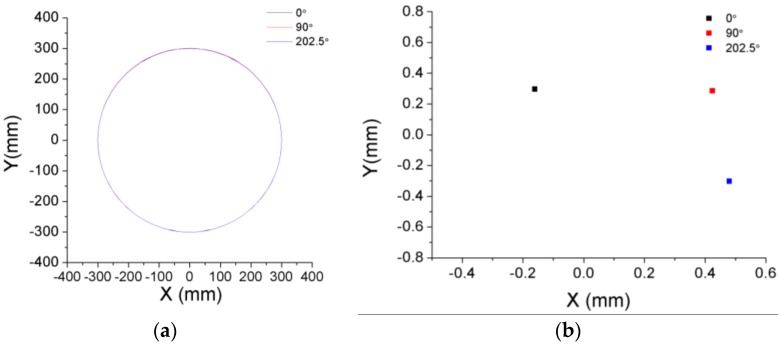
Disk circumferences calculated from the TC measurements of the three OFSs at three different turning positions: 0°, 90°, 202.5° (**a**); Positions of the centers of the circumferences (**b**).

The eccentricity of the disk was determined in three different positions: the TC measurements were acquired with the disk in an initial position of 0° and with the disk rotated 90° and 202.5° with respect to this initial position. Figure 9a depicts the results for the three positions of the disk. As expected, the differences between the circumferences are very small. Their corresponding centers differ from the theoretical one in less than 0.65 mm, as can be seen in Figure 9b. This distance means a shift of the center of about 0.2% of the circumference radius, which makes it quite difficult to distinguish each circumference in Figure 9a.

## 4. Conclusions

The vibrational behavior of a rotating disk has been studied from the tip-clearance measurements provided by three optical fiber sensors. This disk is a model of a real aircraft engine component. It was installed in a wind tunnel, where real working conditions were simulated. Although the sensors were initially designed to perform the TC measurements in low pressure turbines, the configuration was adapted to successfully perform these measurements in the rotating disk. A first prototype of the disk was completely characterized by identifying not only the amplitude and frequency of the vibration, but also the number of nodal diameters of the disk and its variations during the tests. All this valuable information allowed the disk manufacturer to design an upgraded prototype of the disk. This, in turn, was also characterized by examining its vibrational behavior in order to assess the improvements in performance. The obtained results have confirmed a clear improvement in the design of the component. Therefore, the suitability of this innovative optical system both to characterize and to improve rotating disks has also been demonstrated.

## Figures and Tables

**Table 1:** Uncertainty for each sensor obtained in laboratory tests.

Sensor	Uncertainty (µm)
1	7
2	7
3	10

**Table 2:** Results for the first prototype at 0 rpm.

Pressure Difference (bar)	ND	Sensor 1			Sensor 2			Sensor 3		
		Amplitude (µm)	*f_os_* (Hz)	*f_disk_* (Hz)	Amplitude (µm)	*f_os_* (Hz)	*f_disk_* (Hz)	Amplitude (µm)	*f_os_* (Hz)	*f_disk_* (Hz)
2.3	5	2.5	2238	2238	0.2	2238	2238	0.6	2238	2238
2.4	5	20.1	2238	2238	19.3	2238	2238	46.2	2238	2238
2.5	5	57.7	2238	2238	19.7	2238	2238	22.9	2238	2238
2.6	5	87.8	2236	2236	201.4	2236	2236	194.3	2236	2236
2.7	5	79.2	2236	2236	624	2236	2236	171.2	2236	2236

**Table 3:** Results for the first prototype at RPM_nominal_.

Pressure Difference (bar)	ND	Sensor 1			Sensor 2			Sensor 3		
		Amplitude (µm)	*f_os_* (Hz)	*f_disk_* (Hz)	Amplitude (µm)	*f_os_* (Hz)	*f_disk_* (Hz)	Amplitude (µm)	*f_os_* (Hz)	*f_disk_* (Hz)
2.2	5	4	2358	2233	2.2	2358	2233	5.2	2358	2233
2.3	5	15.5	2358	2233	2.2	2358	2233	45.1	2358	2233
2.4	5	33	2357	2232	3.5	2357	2232	32.7	2357	2232
2.5	5	67.4	2122	2247	41.4	2122	2247	58	2122	2247
2.6	4	180.3	1474	1574	105.8	1474	1574	185.7	1474	1574

**Table 4:** Results for the first prototype at 1.5 × RPM_nominal_.

Pressure Difference (bar)	ND	Sensor 1			Sensor 2			Sensor 3		
		Amplitude (µm)	*f_os_* (Hz)	*f_disk_* (Hz)	Amplitude (µm))	*f_os_* (Hz)	*f_disk_* (Hz)	Amplitude (µm)	*f_os_* (Hz)	*f_disk_* (Hz)
2.1	5	0.1	2413	2230	0.1	2413	2230	0.2	2414	2230
2.2	5	28.7	2415	2232	3.7	2415	2232	28.8	2415	2232
2.3	5	42.4	2414	2231	23.9	2414	2231	42.7	2414	2231
2.4	4	100.9	1434	1580	84.1	1434	1580	124	1434	1580
2.5	4	128.7	1433	1579	77.1	1433	1579	136.6	1433	1579

**Table 5:** Results for the first prototype at 2 × RPM_nominal_.

Pressure Difference (bar)	ND	Sensor 1			Sensor 2			Sensor 3		
		Amplitude (µm)	*f_os_* (Hz)	*f_disk_* (Hz)	Amplitude (µm)	*f_os_* (Hz)	*f_disk_* (Hz)	Amplitude (µm)	*f_os_* (Hz)	*f_disk_* (Hz)
1.9	5	0.2	2472	2230	0.2	2472	2230	2.8	2473	2231
2	5	0.3	2472	2230	0.2	2472	2230	0.3	2472	2230
2.1	5	25.5	2473	2231	11.1	2473	2231	19.2	2473	2231
2.2	4	44.8	1394	1588	49	1394	1588	67.2	1394	1588
2.3	4	120.4	1394	1588	89.1	1394	1588	125.2	1394	1588

**Table 6:** Results for the second prototype at 0 rpm.

Pressure Difference (bar)	ND	Sensor 1			Sensor 2			Sensor 3		
		Amplitude (µm)	*f_os_* (Hz)	*f_disk_* (Hz)	Amplitude (µm)	*f_os_* (Hz)	*f_disk_* (Hz)	Amplitude (µm)	*f_os_* (Hz)	*f_disk_* (Hz)
2.7	3	1.1	2024	2024	2.5	2024	2024	0.3	2024	2024
2.75	3	0.8	2024	2024	0.6	2024	2024	1.2	2024	2024
2.8	3	4.6	2025	2025	3	2025	2025	4.1	2025	2025
2.85	3	16.1	2025	2025	16.1	2025	2025	83	2025	2025
2.9	3	55	2025	2025	184	2025	2025	31.6	2025	2025

**Table 7:** Results for the second prototype at RPM_nominal_.

Pressure Difference (bar)	ND	Sensor 1			Sensor 2			Sensor 3		
		Amplitude (µm)	*f_os_* (Hz)	*f_disk_* (Hz)	Amplitude (µm)	*f_os_* (Hz)	*f_disk_* (Hz)	Amplitude (µm)	*f_os_* (Hz)	*f_disk_* (Hz)
2.7	3	83.6	1116	1191	63	1116	1191	67.8	1116	1191
2.75	3	294.6	1114	1189	176.1	1114	1189	248.3	1114	1189
2.9	3	119.3	1118	1193	73.8	1118	1193	81.2	1118	1193
2.95	3	151	1118	1193	98.2	1118	1193	110.1	1118	1193
3	3	179.6	1117	1192	90.5	1117	1192	126.6	1117	1192

**Table 8:** Results for the second prototype at 1.5 × RPM_nominal_.

Pressure Difference (bar)	ND	Sensor 1			Sensor 2			Sensor 3		
		Amplitude (µm)	*f_os_* (Hz)	*f_disk_* (Hz)	Amplitude (µm)	*f_os_* (Hz)	*f_disk_* (Hz)	Amplitude (µm)	*f_os_* (Hz)	*f_disk_* (Hz)
2.5	3	97.1	1085	1195	49.5	1085	1195	80.6	1085	1195
2.6	3	201	1084	1194	122.3	1084	1194	138.9	1084	1194
2.8	3	138	1088	1198	83.5	1088	1198	94.3	1088	1198
2.9	3	272	1086	1196	193.5	1086	1196	175.6	1086	1196

**Table 9:** Results for the second prototype at 2 × RPM_nominal_.

Pressure Difference (bar)	ND	Sensor 1			Sensor 2			Sensor 3		
		Amplitude (µm)	*f_os_* (Hz)	*f_disk_* (Hz)	Amplitude (µm)	*f_os_* (Hz)	*f_disk_* (Hz)	Amplitude (µm)	*f_os_* (Hz)	*f_disk_* (Hz)
2.3	3	95.5	1064	1208	131.5	1063	1207	116.5	1063	1207
2.4	3	162.9	1055	1199	114	1063	1207	117.1	1063	1207
2.6	3	96.1	1059	1203	91	1063	1207	83.6	1063	1207
2.7	3	256.6	1057	1201	206.5	1057	1201	151.2	1057	1201

**Table 10:** Reduction in amplitude and frequency vibration in the second prototype for each optical sensor.

Working Point	Sensor 1		Sensor 2		Sensor 3	
	Amplitude (%)	Frequency (%)	Amplitude (%)	Frequency (%)	Amplitude (%)	Frequency (%)
0 rpm 2.7 bar	98.6	9.48	99.6	9.48	99.8	9.48
RPM_nominal_ 2.6 bar	53.6	24.3	40.4	24.3	63.5	24.3
1.5 × RPM_nominal_ 2.5 bar	24.5	24.3	35.8	24.3	41	24.3
2 × RPM_nominal_ 2.3 bar	20.7	23.9	-47.6	24	6.95	24
